# Study protocol for a randomized single-center cross-over study: Dapagliflozin treatment in recurring kidney stone patients

**DOI:** 10.1371/journal.pone.0322034

**Published:** 2025-04-24

**Authors:** Haris Omić, Michael Eder, Harald Herkner, Christian Seitz, Željko Kikić, Tarek Arno Schrag

**Affiliations:** 1 Department of Medicine III, Division of Nephrology and Dialysis, Medical University of Vienna, Vienna, Austria; 2 Department of Emergency Medicine, Medical University of Vienna, Vienna, Austria; 3 Department of Urology, Medical University of Vienna, Vienna, Austria.; Katholieke Universiteit Leuven UZ Leuven: Katholieke Universiteit Leuven Universitaire Ziekenhuizen Leuven, BELGIUM

## Abstract

**Introduction:**

Urolithiasis is one of the most common diseases worldwide, characterized by high morbidity and significant treatment-related costs, with a rising prevalence of up to 20%. The relapse rate within the first 10 years after initial treatment is estimated to be about 60%. Given the increasing prevalence, healthcare-related costs associated with urinary tract stones in the USA are expected to reach up to US $1.24 billion annually by 2030.

Current prophylactic therapy for urolithiasis recurrence includes lifestyle modifications, citrate supplementation, and pharmaceuticals. However, a high number of cases remain unresponsive to available pharmacological therapies. Though initially developed for the treatment of Diabetes mellitus, SGLT-2 inhibitors have shown promise in decreasing cardiac and renal endpoints across multiple indications. Recent registry studies have indicated that patients receiving SGLT-2 inhibitors exhibit lower rates of urolithiasis incidence, suggesting a potential reduction in recurrence rates and associated mortality.

**Objectives:**

We hypothesize that SGLT-2 inhibitors (Dapagliflozin), owing to their multiple pleiotropic effects, may offer a viable treatment option for the prophylaxis of high-risk calcium oxalate kidney stones and reduce urinary calcium oxalate output.

**Methods:**

This study will proceed in two phases: an exploratory phase and a randomized controlled phase. In the exploratory phase, 22 participants with indications for dapagliflozin treatment will be evaluated before and after treatment initiation to ascertain the concrete effect size regarding oxalate and calcium-sparing effects. This data will inform the calculation of the study sample size (ranging from 17 to 104 participants) to include high-risk calcium oxalate kidney stone formers in a randomized controlled crossover study design. Treatment phases—one with dapagliflozin and one with placebo—will alternate with wash-out phases involving placebo. The primary outcome is the reduction of oxalate excretion in 24-hour urine samples compared to baseline values after 8 weeks of therapy. Secondary objectives include analysing effects on kidney function, the frequency of urolithiasis, and treatment tolerance. Additionally, in-depth metabolomics analyses will explore pathophysiological pathways during treatment. Investigators, patients, and research staff will be blinded to the randomization list. This study was initially registered under EudraCT (Nr:2022-000994-13) and has been transitioned to CTIS (Nr: 2024-519371-25-00) to comply with EU Regulation 536/2014, ensuring streamlined management and transparency.

**Discussion:**

Dapagliflozin’s pleiotropic effects may provide a novel prophylactic treatment option for urolithiasis. This study aims to evaluate potential treatment effects in a prospective RCT and elucidate potential pathophysiological pathways through in-depth metabolomics analyses. SGLT-2 inhibitors have the potential to transform the landscape of urolithiasis treatment, reduce the healthcare burden on individuals and the system, and significantly improve patient quality of life.

## 1. Background

### 1.1 Prevalence

Urolithiasis is one of the most common diseases globally with high comorbidity and high treatment costs. The prevalence ranges from 1% to 20% [1,2], and relapse rates within ten years are approximately 60%. [3] This significant variability is caused by climate, dietary, ethnic, genetic, and geographical factors. Especially in developed countries, the prevalence of urolithiasis is high (>10%) [1,4,5]. Approximately between 25 and 49 million Europeans had a symptomatic kidney stone in 2011 [6]. So far only estimated costs for the USA were calculated. The authors expect the annual costs of urolithiasis to rise from $ 2.81 billion/year in 2000 to an additional $1.24 billion/year by 2030 [7].

The prevalence of urolithiasis has been increasing in the last decades. The “Reykjavik study”, a population-based cohort study from 1967 to 1991, provided valuable insight into the development of urolithiasis. For instance, in the cohort aged 50–59, the prevalence of urolithiasis rose from 4.8% to 6.2% [8]. A reanalysis of the Reykjavik study with prolonged follow-up from 1985 to 2008 documented the dramatic increase in urolithiasis. In both genders, the incidence of asymptomatic kidney stones had a growth rate of 300% in men over the age of 40 and women over the age of 50. This change may be due in part to technical progress in terms of abdominal imaging [9].

There is also evidence of increasing demand for treatment in symptomatic kidney stone patients. Between 2000 and 2010 the data from the Hospital Episode Statistics website, a data service provider of the National Health Service in the United Kingdom, showed an increasing rate of upper tract kidney stones requiring hospital treatment of 63%. The authors explained this increase by lifestyle factors, i.e., adiposity and/or metabolic syndrome, and with the already mentioned technical progress in the stone imaging programs and the increasing diagnostic sensitivity [10]. In the United Kingdom, the incidence of kidney stones among those less than age 75 has been stable at 85,000 cases per year. However, the incidence is increasing for both men and women aged 76 and over [11]. In other European countries with similar dietary habits studies showed an analogous effect of increasing prevalence. In Italy, for both men and women, the prevalence of kidney stone development increased between 1986 and 1998 from 6.8% to 10.1% in men and from 4.9% to 5.8% in women [12]. A rising prevalence from 4.2% to 5.1% was recorded in Spain between 1986 and 2007 [6]. In Germany, the overall prevalence from 1979 to 2001 rose only from 4.0% to 4.7%. However, in patients over age 65, the prevalence increased to 9.5% [2]. In summary, the prevalence of urolithiasis has been rising over the past three decades in both European men and women but currently reliable predictions about future rates cannot be made [6].

### 1.2. Risk factors

There are three fundamental types of risk factors associated with kidney stone formation – the modifiable urinary and dietary risk factors, and the non-modifiable genetic and environmental risk factors. In reality, a combination of different weighting factors is most likely to be operational [13].

#### 1.2.1 Urine composition.

The first group of modifiable factors is usually caused by a chemical imbalance of urine composition that can be induced by several different abnormalities. The degree of influence of the particular abnormalities differs in their impact on forming different types of kidney stones [14]. The risk factors with the greatest influence on calcium oxalate stone formation are high oxalate and high calcium excretion in the urine, plus low citrate excretion and low urinary volume [15]. Levy et al. tracked 1,270 patients with reoccurrence of kidney stone formation and quantified their risk factors over 15 years; they found it was rare for patients to have only a single risk factor. The frequencies of these risk factors within the study population were: hypercalciuria (61%), hypocitraturia (28%), urinary volume < 1.0 L/day (15%), and hyperoxaluria (8%). The data were not adjusted for underlying primary diseases. [16] Another study compared risk factors among patients with a single episode of urolithiasis against those of patients with no history of urolithiasis, targeting urine chemistry. The only significant difference involved urinary citrate and calcium excretion [17].

#### 1.2.2 Diet and fluid intake.

The impact of diet on stone development is still under research, but it has been shown that some dietary factors modify the risk of reoccurrence. A constantly low fluid intake – the exact amount depends on geographical and climatic factors – increases the risk of urolithiasis. It is recommended to avoid urine output of less than 2.0 liters per day, with a urine specific gravity over 1,010, and total sodium intake greater than 5.0 gm per day, as both are associated with increased rates of urolithiasis [18–22]. Furthermore the composition of oral fluids consumed is related to the overall risk. Fluids that diverge from a neutral pH [19], including carbonic acid [20,21] or phosphoric acid [20,21] increase the risk of stone recurrence.

It has also been demonstrated that a variety of eating habits influence the risk of stone formation. A questionnaire survey among 57.446 patients showed that a higher consumption of fresh fruits and wholegrain products showed a lower risk of stone development. A higher risk of kidney stone formation was associated with a higher intake of zinc. Furthermore, a lower risk of kidney stone formation is found among vegetarians compared with meat eaters with an intake of animal-based proteins over 100 g/day [23]. Higher consumption of dietary calcium was associated with a decreased risk of kidney stone formation in multiple large studies including women aged 34–59 (N=91,731, Nurses’ Health Study I) [24], young women (age 27–44, N=96,245, Nurses’ Health Study II) [25] and men (age 40–75, N=45,619) [26]. The underlying hypothesis of this, seemingly counterintuitive association may be related to increased binding of enteral oxalate by dietary calcium. Calcium intake from supplements on the other hand was associated with a small but significant increase in stone formation risk [24] – possibly because of the often-different timing of supplement increase (early in the morning, in the absence of oxalate intake) compared to dietary calcium. Potassium intake was also significantly associated with a reduced stone risk in two [24,26] of the three studies mentioned. Decreased urinary excretion of calcium and a high alkali load in many potassium-rich components may explain this association [27]. Magnesium uptake on the other hand was not significantly associated with stone risk in multivariate analysis [25,26].

#### 1.2.3 Underlying conditions and genetic risk factors.

In addition to the modifiable risk factors there are also non-adjustable risk factors. It has been demonstrated that climate and geographical factors increase the risk of de-novo and recurrent kidney stone formation [28,29]. A positive family history has also been shown to be associated with an almost three-fold increased risk for urolithiasis in the health professional follow-up Study in the USA in 38,000 males [30].

Certain genetic factors seem to predispose to a higher vulnerability for developing calcium-containing kidney stones [30–33]. The genetic influence on risk is still under research, but several affecting genes were found. Halbritter et al. identified 50 mutations in 14 out of 30 researched genes. That way they were able to describe 14.9% of all urolithiasis cases (11% in the adult participants; and 21% in the pediatric participants) in the study cohort. The identified genes are: ADCY10/SAC, AGXT, ATP6V1B1, CLCN5, CLDN16, CYP24A1, SLC22A12, SLC2A9, SLC34A1, SLC34A3, SLC3A1, SLC4A1, SLC7A9 and SLC9A3R1. [34] However, the genetic influence on kidney stone formation is caused by a variety of multilocular genes that affect the absorption, excretion, and resorption of calcium, the absorption and excretion of citrate, and the absorption of oxalate. The protein-biosynthesis products of the involved genes are calcium channels (kidneys and GI-tract), calcium-sensing receptors, tight junction proteins, oxalate exchangers, phosphate transporters as well as vitamin D- receptor, and -24-hydroxylase. So far only a few single-nucleotide polymorphisms (SNP) seem to be associated with a higher hereditary kidney stone risk. The concept of polygenic risk scores has been recently introduced by Paranjpe et al; they found a risk score based on multilocular genes that predicts a higher risk of kidney stones even in the absence of typical clinical risk factors [33].

Urolithiasis is often embedded in a wide spectrum of comorbidities that are associated with a risk of kidney stone formation such as primary hyperparathyroidism [35], hypertension [36,37], gout [38, 39], diabetes mellitus [40, 41], obesity [42–45], medullary Sponge kidney [46], cystinuria [47] and distal tubular renal acidosis [48]. Furthermore, a variety of health conditions such as a history of bariatric surgery [49], asymptomatic [50], and symptomatic infections of the urinary tract also have an increased risk for stone formation [51].

### 1.3 Health-economic burden

From an economic point of view, urolithiasis keeps producing higher costs every year. In the USA the costs of caring for patients with nephro- and/or urolithiasis rose 50% from 1994 to 2000. 53% of these costs were caused by outpatient care. The total costs in 2000 were estimated at US $2,1 billion (US $971 million for inpatients, US $607 million for outpatients, and US $490 for emergency service) [52]. Based on the increasing prevalence of risk factors like obesity, diabetes, and the growing population, an increase of healthcare costs associated with stones in the urinary tract are expected to increase by US $1.24 billion every year by 2030 [7].

#### 1.3.1 Calcium-oxalate stones and recurrence rates.

Urolithiasis is one of the most common diseases globally with high comorbidity and treatment costs with a prevalence from 1% to 20% [1, 2]. The relapse rate of calcium-oxalate stones within the first 5 years after initial treatment is about 40% and increases to 75% in the first 20 years. Several studies demonstrated a recurrence rate between 43–80% at three years among a cohort of subjects with a history of recurrent calcium stone formation [53–60].

#### 1.3.2 Preventive measures for recurrent kidney stones.

The current basis for the conservative treatment of recurrent kidney stones is lifestyle modifications, citrate supplementation, and pharmaceuticals. After the first episode of kidney stones lifestyle modification is recommended to every patient. Those with low-risk factors for recurrence do not require medication. Lifestyle modification alone is sufficient. High-risk patients require additional preventive measures including supplements and drug treatment adjusted to their metabolic situation [18–21,61].

### 1.4 . General measures

The general cornerstones of the prevention of recurring kidney stones are increasing the daily water intake, introduction of a healthy diet, and lifestyle adjustments including body weight reduction and increased physical activity. Increasing the total fluid intake to a level between 2.5 and 3.0 L per day is the second most crucial step in the prevention of urolithiasis. This measure is meant to achieve an ensured diuresis of 2.0 to 2.5 L per day with a specific gravity below a level of 1.010 [18–22]. The fluid intake is meant to be consumed consistently throughout the day and only liquids with a neutral pH should be consumed [19]. It is especially important to avoid carbonated and phosphorous-containing drinks as this measure has been proven to decrease the risk of recurrent urolithiasis [20,21]. According to the EAU Guidelines of 2023 the increased intake of fluids with a recommended urine volume of 2.0–2.5 L/ day shows level 1a evidence in the prevention of recurrent stones in the urinary tract [62].

A balanced diet including a high ratio of vegetables and fibers, a low-fat diet, a calcium intake of about 1.0 to 1.2g per day, a reduced intake of salt up to a maximum of 3.0 to 5.0g per day, and an intake of animal proteins of 0.8 to 1.0g per kg per day may reduce stone development risk [18,19,62]. Furthermore, patients are strongly advised to reduce or maintain a body weight to a BMI< 25 kg/ m² and follow a healthy way of activity [18,19,61].

According to the EUA guidelines 2023, especially patients with a high risk of hyperoxaluric stones are advised to lower the ratio of oxalate in their food. Patients with a history of hyperuricosuric stones should consume a reduced daily amount of purine [62]. Even though the evidence of lifestyle modification as prevention for the development of urolithiasis is limited in some instances, comorbidities that could increase the risk of new stones like cardiovascular disease or diabetes can also be prevented by these same lifestyle changes [62].

#### 1.4.1 Alkaline citrate supplementation.

Citrate supplementation in combination with lifestyle modification is an effective preventive treatment for hypercalciuric and hypocitraturic patients. The EAU Guidelines of 2023 recommend alkaline citrates, as monotherapy or in combination with thiazide diuretics to decrease the risk of recurrent stone formation (level 1a) [62].

Also, patients suffering from enteric hyperoxaluria as a result of a variety of gastrointestinal disorders can be treated with alkaline citrate to reduce the risk of stone formation [62]. Potassium citrate is the most commonly supplemented salt; the recommended dosage is 5 to 12g per day [18–20]. An alternative agent in usage is sodium citrate, but it may cause increased secretion of sodium and calcium, which is the opposite of the desired result [18–20].

#### 1.4.2 Drug treatment.

There are three types of pharmaceutical agents used as a preventive treatment for recurring calcium stones only – thiazide diuretics, allopurinol, and febuxostat – but only associated with an additional lifestyle modification as previously mentioned [62].

Thiazide diuretics, most frequently hydrochlorothiazide (HCT), as prevention therapy for calcium-oxalate kidney stones has been used for the last decades, but only when used at high dosages in hypercalciuric patients (level 1a). The EAU guidelines of 2023 recommend a daily dosage of 25–50mg [62]. But, the described dosages may lead to more frequent side effects, including hyponatremia and hypotension [20,21]. However, the NOSTONE trial recently disproved its efficiency. The data showed no linear relationship between therapy with HCT and the incidence of the reoccurrence of urolithiasis (risk of reoccurrence without therapy: 0.45; HR of 12,5mg HCT/d: 0.90; HR of 25mg HCT/d: 0.65; HR of 50mg HCT/d: 0.50). Even more, observed symptomatic urolithiasis event rate was higher than the expected [63]. Furthermore, long-term therapy with HCT increases the risk of melanoma and non-melanoma skin cancer in non-Asian countries leading to HCT being no longer recommended by the guidelines of 2023 of the German Urology Association as prevention therapy for recurrent kidney stone patients. A substantial alternative has so far not been found.

For hyperuricosuric subjects allopurinol or febuxostat is recommended. No additional preventative effects were shown with combination therapy (thiazide diuretics + allopurinol, thiazide diuretics + alkaline citrates, allopurinol + alkaline citrates) compared with appropriate monotherapy [18–21].

However, the evidence for stone-preventing therapies based upon the determining stone composition or on biochemistry measures is low or no longer recommended. Furthermore, especially in high-risk patients, the outcome of the above-mentioned therapies varies widely and has a significant failure rate [21,62]. Therefore, further research is needed to identify effective therapy options for patients with a high risk of reoccurrence based on a significantly increased risk of and/ or an increased number of urolithiasis events within the past years. If successful, decreasing the frequency of recurrent nephrolithiasis would increase the quality of life for these patients and reduce morbidity and healthcare-related costs.

#### 1.4.3 SGLT-2 inhibitors.

Though initially developed for the treatment of diabetes, SGLT-2 inhibitors have been found useful for many additional indications due to the pleiotropic effects observed in multiple studies. This includes treatment of heart failure, and more recently, chronic kidney disease. In 2020 the randomized multicentre DAPA-CKD study analysed the effect of dapagliflozin with a daily dosage of 10mg compared to a placebo in 4304 patients with chronic kidney disorder and with and without diabetes mellitus II. All patients had already received therapy with ACE inhibitors or angiotensin-receptor-blockers for a minimum period of 4 weeks. Primary endpoints were defined as a permanent increase in kidney function of more than 50%, new onset of Stage IV chronic kidney disease, or mortality due to renal or cardiac disorders. Inclusion criteria were a urine protein-creatinine ratio ≥ 200 and ≤ 5,000mg/g and an eGFR ≥ 25 and ≤ 75mL/min/1.73m^2^ [64].

Within an interim analysis, the phase III study was recommended to be stopped after a median follow-up of 2.4 years because of highly significant positive effects. The primary endpoint was reached by 197 patients of the dapagliflozin group and by 312 patients of the placebo group (HR: 0.61; 95% KI 0.51–0.72; p = 0.00000000028); under dapagliflozin therapy, there was no difference detected in the superiority between patients with or without diabetes. Furthermore, dapagliflozin significantly reduced all secondary endpoints: 1) deterioration of the kidney function or death due to loss of kidney function (HR: 0.56; 95% KI 0.45–0.68; p < 0.0001); 2) hospitalization due to cardiac insufficiency or death due to cardiovascular reasons (HR: 0.71; 95% KI 0.55–0.92; p = 0.0089); 3) total mortality (HR: 0.69; 95% KI 0.53–0.88; p = 0.0035) [64].

The findings show evidence of a positive effect of dapagliflozin on patients with CKD to stabilize or improve kidney function regardless of pre-existing diabetes and/or cardiac insufficiency. With regards to risks and side effects, there was no difference detected between dapagliflozin and the placebo [64].

#### 1.4.4 SGLT-2 inhibitors and urolithiasis.

The current literature provides multiple examples of potential treatment effects of SGLT-2 inhibitors in urolithiasis patients. In clinical trials, it has been shown that SGLT-2 inhibitors – the group of drugs dapagliflozin belongs to – lower serum uric acid [65–67]. A prospective register study by Kristensen et al. with more practically oriented findings included 12,325 patients in both the SGLT-2 inhibitors cohort and the GLP-1 receptor agonists cohort. The incidence of urolithiasis in the SGLT-2 inhibitor cohort was only 49% of the incidence among the GLP-1 receptor agonist cohort. This difference was statistically significant. The incidence of urolithiasis in the SGLT-2 inhibitors cohort was 50% lower compared to the GLP-1 receptor agonists cohort [2.0 per 1,000 person-years vs. 4.0 per 1,000 person-years); the overall incidence difference was 1.9 per 1,000 person-years (95% CI -2.8 – (-1.0)] (HR 0.51; 95% CI 0.37–0.71).

Moreover, 731 patient-pairs with a history of nephrolithiasis and similar baseline characteristics suffered from recurrent urolithiasis; the recurrence-free time was a little longer in the SGLT-2 inhibitor cohort (1,485 vs 1,386 person-years). The incidence of a case of recurrent nephrolithiasis was 36 per 1,000 person-years in the SGLT-2 inhibitor group and 53 per 1,000 person-years in the GLP-1 receptor agonist group. The difference in incidence was -17 per 1,000 person-years [95% CI -33 – (-1.5)] (HR 0.68; 95% CI 0.48–0.97) [68].

Another epidemiological trial targeting the effects of SGLT-2 inhibitors on the nephrolithiasis frequency screening data of 628,570 women 909,628 men and was performed in Japan. They showed a significantly decreased prevalence in men with diabetes mellitus treated with SGLT-2 inhibitors vs. men without one (2.28% vs. 2.54%; odds ratio: 0.89; 95%-CI:0.86–0.94). This difference could be shown over all ages except the men aged 80 years and older [69].

Furthermore, Anan et al. showed in a rat model the connection between SGLT-2 and nephrolithiasis. They induced calcium oxalate stones by administering ethylene glycol and showed a reduced expression of the SGLT-2 gene and protein as well as an increased level of Osteopontin (OPN] in the cells of the proximal tubules; by adding Phlorizin (predecessor of SGLT-2 inhibitors] the expression of SGLT-2 re-increases and the OPN level decreases. High OPN expression rates lead to calcium oxalate stones-induced damage to the tubules while an additional SGLT-2 inhibitor decreased OPN and reduced the rate of these damages [69].

But OPN in its role as proinflammatory cytokine and effector on the macrophage response [70] in combination with the activation of the transforming growth factor beta (TGFb) pathway is crucial for nephrolithiasis formation and tubular fibrosis [71–75]. In the trial of Anan et al., they found a large turnover of a variety of inflammation participants as well as macrophage infiltration in the cortical interstitial space in the kidneys of the ethylene glycol-treated rats. All the described effects, the high OPN levels, and the intestinal fibrosis following the macrophage infiltration decreased under phlorizin treatment.

Focusing the urine output they showed that the urinary oxalate output between the rats that were stone-induced with ethylene glycol and those that were induced and received an SGLT-2 inhibitor was around one standard deviation (Oxalate in mg/24h urine (mean ± SD): 10.7 ± 0.6 vs. 9.8 ± 1.0). They concluded that SGLT-2 inhibitors have a preventing effect on calcium oxalate stone formations and decrease inflammatory pathways leading to reduced kidney injury and fibrosis [69].

In synopsis, it has been shown that SGLT-2 inhibitors can reduce the urolithiasis frequency over all genders and age groups and they can - lower the oxalate output and the inflammatory pathways in the kidneys with high stone risk. In combination with the shown fact that a reduced calcium oxalate urinary output is associated with a reduced urolithiasis risk and reoccurrence risk, [76] we expect that dapagliflozin can reduce the urinary oxalate/ and or calcium excretion in recurrent urolithiasis patients. We further anticipate that this effect will increase the quality of life and decrease the rate of reoccurrence of the affected patients and the related morbidity and healthcare burden.

### 1.5 Need for new treatment options

The large individual and economic burden upon the health care system demands fundamental changes in the treatment and prevention of urolithiasis. The recommended lifestyle changes including increasing fluid intake, weight loss, reduction of sodium consumption, and increase of potassium-rich foods are difficult to adhere to and often only of short benefit. For instance, even under the conditions of an RCT where intensive coaching was applied to increase water intake, the treatment effect was mild with an increase of urinary volume of 0.6 L/day in the treatment group [77]. Similar findings have been observed in pragmatic RCTs aiming at weight reduction through structured coaching on behavioral changes [78].

The current drug treatment options are limited by tolerance, side effects, and low efficacy of therapy. Therapy with HCT has been shown to have no benefit compared with the placebo group in the frequency of events nor in the risk of reoccurrence independently of the dosage. Additional to the lack of efficacy in this specific indication it should be emphasized that long-term therapy with HCT increases the risk of melanoma and non-melanoma skin cancer in non-Asian countries, and regular screening for this skin cancer is necessary [79]. Due to this fact, HCT is no longer recommended by the guidelines of 2023 of the German Urology Association as prevention therapy for recurrent kidney stone patients. Alkaline citrate supplementation may provide beneficial effects in hypocitraturia, but its application is limited by reduced gastrointestinal tolerance, leading to treatment discontinuation in a large proportion of patients.

In summary, in the current situation, there exists no relevant and effective therapy options for the mass of patients with a high risk of reoccurrence based on a significantly increased risk of and/ or an increased number of urolithiasis events within the past years. Further research is needed to identify at least one option for this specific group of patients. If successful, decreasing the frequency of recurrent nephrolithiasis would increase the quality of life for these patients and reduce morbidity and healthcare-related costs.

## 2. Materials and methods

### 2.1 Study aim and perspective

In summary, further research is needed to confirm the assumption that Dapagliflozin can reduce oxalate and/or calcium excretion and in further consequence reduce the risk and frequency of urolithiasis. Moreover, the real pathophysiological effects are unknown, which is why we also include preliminary and in-depth metabolomics analysis of blood and urine- to narrow down potential biochemical pathways.

As one of the most common diseases globally, urolithiasis has a prevalence of 1% to 20% combined with high comorbidity and treatment costs [1,2]; the relapse rate within the first 10 years after initial treatment is about 60% [3]. After the first episode of kidney stones lifestyle modification is recommended to every patient. Supplementation and pharmaceuticals are recommended to be reserved for recurrent episodes. High-risk patients need preventive therapy adjusted to their metabolic abnormalities [18–21,61].

The evidence for current stone-preventing therapies based upon the determining stone composition or on biochemistry measures is low. Furthermore, especially in high-risk patients, the outcome of the above-mentioned therapies varies widely and has a significant failure rate [21,62]. Recent studies focusing on urolithiasis event-rates however showed a reduction of the frequency [68, 69]. In the case that dapagliflozin decreases the incidence of urolithiasis regardless of underlying comorbid disease a serious alternative to repetitive surgical treatment can be offered to affected patients. Especially hyperoxaluric patients will benefit from the new case application of dapagliflozin concerning reduced urolithiasis risk.

### 2.2 Trial design

*Study type:* Pharmacological randomized cross-over single-center study

Our study will be the first one prospectively targeting the effect of dapagliflozin on urolithiasis and the urinary compounds playing a role in its development we chose a two-part design to target relevant problems: Since no comparable, prospective trials in humans have been performed there is no data about the expected effect size. Therefore, the upstream exploratory phase will be performed to generate prospective data that will be the base of the sample size calculation of the main trial. (see 2.3 Sample size calculation).

#### 2.2.1 Exploratory phase.

The exploratory phase will focus on metabolomic analyses to identify the effects of dapagliflozin therapy in humans on the urine composition respectively electrolytes and small molecules that may interfere with the urolithiasis frequency such as oxalate, calcium, magnesium, citrate, and uric acid.

Therefore, one group of 22 participants with a diagnosed cardiomyopathy and/or diabetes Mellitus and indication for dapagliflozin will get a daily oral administration of 10mg dapagliflozin combined with regular blood- and urine test to generate data for the calculation of the effect size. Furthermore, we hope to gain more information about the pharmacologic mechanisms of how dapagliflozin modifies the frequency of urolithiasis events in humans as it has been shown in earlier studies [64,68,69].

**Fig 1:**
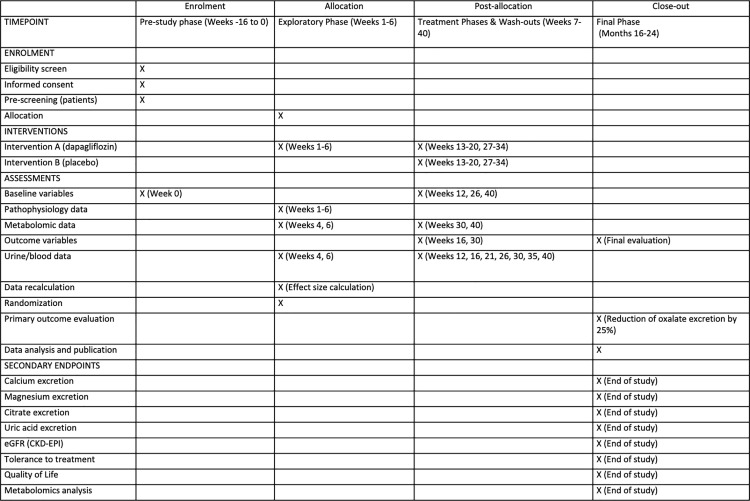
SPIRIT-Study Workflow and Assessment Timeline with Primary and Secondary Endpoints.

The SPIRIT-Study workflow and assessment timeline with primary and secondary endpoints have been presented in Fig 1 and S1. Table.

#### 2.2.2 Randomized controlled trial.

The second part, the main trial will also be a randomized cross-over single-center study in patients with a high risk of urolithiasis reoccurrence to show if dapagliflozin can modify the urine composition in hyperoxaluric patients with a high stone-forming risk. After a recalculation of the sample size based on the findings of the exploratory phase screenings of potential participants will run to gain the population needed for this trial section.

We hope to demonstrate that dapagliflozin is a new therapy option for high-risk stone formers with no known secondary causes. Participants eligible for the main trial must meet two key criteria: first, they must be selected from a group of patients with similar clinical profiles, specifically those classified as high-risk stone formers who have experienced a significantly increased frequency or severity of urolithiasis events in recent years. Second, they must not have any identifiable secondary causes for these events, such as known metabolic disorders, anatomical abnormalities, or medications known to predispose to stone formation (see 2.4 Trial participants).

After the end of the main trial, the participants of the main trial will be offered to be included in a follow-up trial. The participants will continue to get a daily oral dosage of 10mg dapagliflozin for a period of up to two years. By that, we would like to demonstrate the effect of dapagliflozin on the urolithiasis event rate in humans in the first prospective trial even if short-term or metabolomic data of the first two study phases will gain no new information about the pathophysiology and the pharmacologic pathways behind this disease.

### 2.3 Sample size calculation

#### 2.3.1 Sample size calculation of the exploratory phase.

As mentioned above, no data exists about the effect of dapagliflozin on the oxalate and calcium 24-hour urine excretion. Given the observed decrease in urolithiasis event rates in previous studies [68,69] and findings from a rat model investigating the impact of SGLT-2 inhibitors on urolithiasis [69] we anticipate a 10%-50% reduction in 24-hour urinary excretion of oxalate and calcium compared to placebo. This expectation is supported by evidence suggesting that a 10% decrease in urinary excretion of calcium and oxalate is linked to a significant 8% reduction in the risk of recurrence [76]. We, therefore, expect a) the effect size of dapagliflozin to be a reduction of urinary excretion of lithogenic molecules between 15% and 25% reduction and b) subsequently to have a clinically relevant potential to lower the risk of urolithiasis reoccurrence.

The sample size calculation is based on our primary outcomes of the intraindividual comparisons. The sample size calculation based on a paired t-test was performed using G*Power Version 3.1.9.4. The significance level of two-sided testing was set to 0.025 to acknowledge the two primary endpoints [80]. We set the power to 90% and the alpha error to 5%. Due to no comparable data, we assume a standard deviation of 20% and a correlation between the intraindividual comparisons of 0.1. Based on the sample size models in Table 1. (assumed effect size of 25%, correlation between the groups of 0.1) we proclaim a group size of 18 participants plus an expected dropout rate of 4 so a total of 22 participants is reasonable.

**Table 1:** potential group size calculations based on earlier findings.

Mean difference	Mean ± SD group 1	Mean ± SD group 2	Group size	Group size + 20% dropout
10%	1.0 ± 0.2	1.1 ± 0.22	86	104
15%	1.0 ± 0.2	1.15 ± 0.23	42	51
20%	1.0 ± 0.2	1.2 ± 0.24	26	32
25%	1.0 ± 0.2	1.25 ± 0.25	18	22
30%	1.0 ± 0.2	1.3 ± 0.26	14	17

#### 2.3.2 Sample size calculation of the main trial.

Under the Null hypothesis that dapagliflozin reduces the oxalate concentration in the 24h urine by the effect shown in the exploratory phase compared to the baseline value, we will re-calculate the needed number of participants to demonstrate this effect with 90% power and a 5% alpha error in a two-sided test. In expectation of dropouts an additional number of patients of 20% of the calculated sample size will be added to the study collective.

The sample size will be calculated by using G*Power Version 3.1.9.4 with the following parameters: two-sided t-test, expected effect size 25%; significance level = 0.025; power = 0.9; alpha error 5% dropout rate 20%.

### 2.4 Trial participants

#### 2.4.1 Trial participants of the exploratory phase.

We plan to include a group of 22 persons in the exploratory phase recruited out of participants suffering from cardiomyopathy and/ or diabetes mellitus. By that, no harm must be feared and especially the diabetic and cardiomyopathy participants will have an indication and a proven benefit of dapagliflozin. Provided that the participants will not suffer any effects pharmaceutical agents that have the potential to interfere with dapagliflozin such as thiazide diuretics, alkaline bicarbonate, bisphosphonate, calcium supplementation, carbonic-anhydrase inhibitors, denosumab, glucocorticoids, loop diuretics, teriparatide, topiramate, xanthin-oxidase inhibitors or 1,25-OH-Vitamin D will be paused four weeks prior of the study to prevent data corruption by through interferences.

#### 2.4.2 Trial participants of the main trial.

As mentioned earlier, the exact sample size will be calculated out of the data of the interim analysis (see 2.3.2 Sample size calculation of the main trial). We plan to include two equal groups in an RCT design. Like above, pharmaceutical agents that have the potential to interfere with dapagliflozin will be paused four weeks prior to the study.

#### 2.4.3 Inclusion criteria.

1. The patient informed about the study procedures as described and documented by the signed consent form
2. Age >18 years
3. Calcium-oxalate stone formers with a high risk of reoccurrence are defined as:
  A. At least two symptomatic or surgically treated kidney stones within the last 10 years; In particular; patients who have experienced at least two distinct symptomatic or surgically treated kidney stone episodes within the last 10 years, confirmed by imaging or surgical retrieval. Each episode must be separated by a period where the patient was either stone-free or had no symptoms, as verified by clinical evaluation or imaging to exclude the possibility of residual fragments from previous procedures, and/or
  B. Single stone kidney formers with risk factors include:
    a. Positive medical family history on kidney stone formations of at least one blood-related relative in the first degree or at least two blood-related relatives in the second degree and/ or
    b. The onset of kidney stone formations within the third life decade or earlier and/ or
    c. Metabolic syndrome and/ or
    d. Obesity (BMI ≥ 30 kg/m²)

#### 2.4.4 Exclusion criteria.

1. Age < 18 years
2. Secondary causes of reoccurrence of nephrolithiasis such as:
  A. Active malignancy
  B. Active or chronic infection of the urinary tract
  C. Bowel diseases (chronic diarrhea, chronic pancreatitis, inflammatory bowel disease), bariatric or intestinal surgery leading to a bowel shortened length of the bowel
  D. Chronic kidney disease (defined as eGFR < 30 ml/min/1.73 m^2^ for more than three months)
  E. Cystic fibrosis
  F. Cystinuria
  G. Eating disorders (anorexia or bulimia)
  H. Hyperuricosuria with or without the necessity of uric-acid lowering therapy, three or more episodes of gout arthritis within the last year before inclusion into the trial
  I. Hypokalemia at first study-ambulance visit (defined as blood potassium level < 3 mmol/L)
  J. Hyponatremia at first study-ambulance visit (defined as blood sodium level < 125 mmol/L)
  K. Neurogenic bladder
  L. Obstructive uropathy or anatomical abnormalities leading to a decreased urine flow if not treated to a fully unblocked flow of urine
  M. Osteoporosis
  N. Polycystic kidney disease
  O. Primary hyperoxaluria
  P. Primary hyperparathyroidism
  Q. Sarcoidosis
  R. Transplanted kidney
  S. Renal tubular acidosis
3. Currently running of prior (within three months) participation in other interventional clinical studies
4. Not fully understood or signed consent form, restrictions leading to inability to understand and follow the procedure as given in the protocol
5. Known allergy to at least one substance used in the trial

### 2.5 Hypothesis and Endpoints

#### 2.5.1 Null hypothesis of the main trial.

Dapagliflozin therapy is not associated with a reduction of oxalate excretion in 24-hour urine by at least 25% compared to the baseline value in hyperoxaluric patients with a high risk of urolithiasis reoccurrence.

#### 2.5.2 Alternative hypothesis of the main trial.

Dapagliflozin therapy is associated with a reduction of oxalate excretion in 24-hour urine by at least 25% compared to the baseline value in hyperoxaluric patients with a high risk of urolithiasis reoccurrence.

#### 2.5.3 Primary endpoint of the main trial.

As the primary endpoint, we chose *the reduction of the urine oxalate excretion after 8 weeks of dapagliflozin therapy* compared to the baseline value that was collected in the washout phase before the treatment period.

As a secondary objective, we chose to compare the oxalate and calcium-sparing effects such as the kidney function, the frequency of urolithiasis events within the study period, and the tolerance to each treatment. The rationale behind this is a combination of two findings: 1) the reduced urolithiasis event rate under SGLT-2 inhibitor therapy in former trials [68, 69] and 2) the reduced oxalate excretion in a rat model about the effect of SGLT-2 inhibitors on urolithiasis [69]. Further, AstraZeneca holds a patent for the use of SGLT-2 inhibitors to prevent and treat kidney stones [81].

#### 2.5.4 Secondary endpoints of the main trial.

- Excretion of calcium in 24h urine at seven-time points before and after SGLT-2 inhibitor therapy
- Excretion of magnesium in 24h urine at seven-time points before and after SGLT-2 inhibitor therapy
- Excretion of citrate in 24h urine at seven-time points before and after SGLT-2 inhibitor therapy
- Excretion of uric acid in 24h urine at seven-time points before and after SGLT-2 inhibitor therapy
- eGFR (CKD-EPI) before and after therapy
- Tolerance to treatment
- Quality of Life Assessment
- *Metabolomics* analysis out of blood and urine samples at four time points before and two-time points after dapagliflozin therapy.

#### 2.5.5 Further Study Procedures.

Prospective collection of baseline variables

- Age
- Number and compositions of previous stone formations
- Composition of last stone
- Diabetes disease type I and II
- Therapy tolerability
- Quality of life assessment using the SF36 questionnaire

#### 2.5.6 Metabolomics analysis.

The metabolomics analysis in this study focuses on specific metabolites and metabolic pathways derived from blood and urine samples. From blood samples, the analysed pathways include glyoxylate and dicarboxylate metabolism, glycine, serine and threonine metabolism, glutathione metabolism, phenylalanine metabolism, beta-alanine metabolism, the citrate cycle (TCA cycle), and several others (full list provided in the supplemental materials). From urine samples, the analysis includes amino acids (e.g., α-aminobutyric acid, isoleucine, methionine, phenylalanine, valine), creatinine, citrate, glycine, hippurate, and additional metabolites. The complete list of planned analyses has been provided in S2. Appendix.

### 2.6 Study schedule

We plan to do blood- and 24-hour-urine screenings including oxalate and citrate as well as metabolomics tests out of blood and urine samples at crucial points of time of the study to get baseline and therapeutic data of the participants. The primary endpoint is confirmatory and is the reduction of oxalate in the *Verum* vs. Placebophase, i.e., Dapaglifozine vs. placebo which will be compared with a two sided-t-test.

#### 2.6.1 Exploratory phase.

Before the main trial is about to begin there will be an exploratory part of the study. 22 participants with a diagnosis of cardiomyopathy, a given indication for dapagliflozin, and that as well might benefit from the therapy, will be included in one group. For 6 weeks all patients will get an oral therapy of 10mg dapagliflozin daily. To get baseline data of all participants blood and urine samples will be collected before the beginning of the exploratory phase; the treatment response will be evaluated by blood- and urine tests after 4 and 6 weeks of therapy. With the collected data an interim analysis targeting the pathophysiology of urolithiasis as well as the effect mechanism of dapagliflozin will be calculated. Only if the data of the exploratory phase shows a positive effect of dapagliflozin on the excretion rate of oxalate, calcium, phosphate, and uric acid the next part of the main trial will begin.

Patients who already participated in the exploratory phase won’t be treated differently and will have to run through all the following phases whether they already received the trial treatment or not—that way the blinding is still given and whether participants nor investigators can extrapolate their status.

#### 2.6.2 Main trial phase.

The main trial will start with wash-out phase I. All patients will receive blood- and 24-hour-urine screenings in week 6 (respectively week 12 overall) to get baseline data. In preparation for the treatment phase I all participants will receive a daily oral placebo administration over the whole period and will be randomized in two groups.

In week 13 the treatment phase I will start and group 1 will receive oral therapy with 10mg of dapagliflozin while group 2 will get an oral placebo administration daily; the treatment response will be evaluated by blood- and urine test after 4 weeks of therapy (respectively week 16 overall) and in the beginning of in washout phase II (respectively week 21 overall). The same test as before will be performed after 6 weeks (respectively week 26 overall) to gain baseline data for treatment phase II. In preparation for the treatment phase II, all participants will receive a daily oral placebo administration. will receive a daily oral placebo administration.

In the end, we will perform treatment phase II with an oral administration of 10mg of dapagliflozin for group 2 and daily placebos for group 1. The treatment response will be evaluated by blood- and urine tests after 4 weeks of therapy (respectively week 30 overall). Finally, the same tests as before will be performed in wash-out phase III in weeks 1 and 6 (respectively weeks 35 and 40 overall). This way we will get data of the non-therapy periods and of the outcome that will be caused by the dapagliflozin therapy. For each patient, a comparison will be made between the excretion of the electrolytes and molecules of the primary and secondary endpoints under dapagliflozin therapy vs. oral placebo administration.

Throughout the whole study procedure, all participants will be advised to follow dietary instructions as recommended in the urolithiasis guidelines of 2023 [62]. The requested measures will include a balanced diet including a high ratio of vegetables and fibers, a low-fat diet, a calcium intake of about 1.0 to 1.2g per day, a reduced intake of salt up to a maximum of 4.0 to 5.0g per day and an intake of animal proteins of 0.8 to 1.0g per kg per day may reduce stone development risk. Furthermore, patients are strongly advised to not lose or gain weight tremendously throughout the trial process so that the date will be comparable over the whole trial period.

By that measure the investigators want to create a comparable environment for all participants that will lead to comparable data once between the data of one participant over the whole trial period and secondly between all participants independently of their individual dietary habits. In combination with a longer observation period variability should be reduced and the gained data will show a realistic representation.

#### 2.6.3 Randomization and stratification.

We will use computer-based randomization without stratification using randomized blocks of small size and permutation of treatments within each block. Investigators, patients, and research staff will be blinded to the randomization list. Due to a secondary sample size calculation, the final randomization will be executed after the final sample size calculation.

### 2.7 Ethical concerns/ risk assessment

We plan a prospective pharmacological randomized cross-over single-center study to find out the effectiveness of dapagliflozin in high-risk patients with recurrent kidney stones. The current preventive therapies previously discussed vary widely and have low to zero success rates in many cases. [21,62,63]. The prospective collection and evaluation of blood and urine samples will determine the efficacy of dapagliflozin for this special group of patients. The performance of our study will be according to the principles of the Declaration of Helsinki 2008. The study has received approval from the Institutional Review Board of the Medical University of Vienna (1205/2022).

The planned study procedures in the current prospective observational project are all embedded in the standard of care procedures. At the study visits samples of blood and urine specimens (approx. 60 ml and 9 ml) will be collected to show if dapagliflozin therapy will produce results in this specific group of patients; that do not pose a potential harm to the study participants. However, we assume that no participant will be subjected to any potential harm related to their participation in the study. No interventional procedures will be performed if not clinically indicated. Due to the assumption that dapagliflozin will reduce the oxalate output, there is a probability that participants will experience a significant benefit from the study project. The results from this trial however have the potential of adding significant insight into the efficacy of dapagliflozin therapy in patients with a high risk of recurrent urolithiasis. These results potentially could be highly valuable for future patients in improving their quality of life, kidney function, and kidney stone-related mortality. This study was initially submitted to the EudraCT database under the identifier 2022-000994-13. However, per the regulatory transition requirements, the study has been submitted to the Clinical Trials Information System (CTIS) under the identifier 2024-519371-25-00. This ensures compliance with the latest clinical trial regulation (Regulation (EU) No 536/2014), which mandates the migration of EudraCT studies to CTIS for harmonized trial management and transparency. Both identifiers are provided for reference and traceability.

#### 2.7.1 Sex- and gender-specific aspects.

The prevalence of urolithiasis in Western countries is higher in men than in women. In the majority of cases, this is most likely related to previously mentioned lifestyle factors and underlying diseases. In all genders, an increasing prevalence has been detected in recent years.

In the application of Dapagliflozin, no significant sex- or gender-specific aspects related to side effects, response, outcome or any other issue have been described. Therefore, an age and sex-balanced patient population will be recruited for our study to produce data that most accurately will assess the relevant variables as determined by previous studies.

Females of childbirth potential are allowed to participate in this clinical trial if they declare to not try to actively become pregnant while participating in this study. There will be no obligatory contraception for these females. Pregnancy tests immediately before study inclusion and at least monthly during the study will be performed.

#### 2.7.2 Study discontinuation and management of serious adverse events (SAEs).

The study will be discontinued for any participant in the event of a serious adverse event (SAE), significant protocol deviations, or if continuation poses a risk to the participant’s health. Study discontinuation may also occur at the request of the participant or their treating physician. Criteria for discontinuing a participant’s involvement in the study include:

- Occurrence of any SAE related to dapagliflozin or placebo that is life-threatening, results in hospitalization, or causes significant disability (diabetic ketoacidosis, severe urinary and genital infections, hypotension, acute kidney injury, fractures, and hypoglycemia).
- Persistent non-compliance with the study protocol, such as failure to attend required study visits or adhere to the treatment regimen.
- Development of any new medical condition or complication that contraindicates continued participation, such as a significant decline in renal function (eGFR <15 ml/min/1.73 m²) or other serious medical conditions requiring immediate treatment outside the scope of the study.
- At the participant’s request, for any reason, or if the participant becomes pregnant during the study period.

In the event of an SAE, immediate steps will be taken to ensure the participant’s safety, including withdrawal from the study treatment if necessary. All SAEs will be reported to the Institutional Review Board (IRB) within 24 hours of awareness, followed by a detailed report within seven days. If necessary, the IRB can recommend study suspension or termination depending on the frequency and severity of the events.

#### 2.7.3 . Informed consent form (ICF).

Before joining this study, all participants will be asked to review and sign an informed consent form. This process is designed to ensure that each participant:

- Understands Why the Study is Being Conducted: Participants will receive clear information about the study’s goals, what we hope to learn, and what participation will involve.
- Knows Participation is Voluntary: Participation in this study is entirely voluntary. Patients will be reassured that they are free to withdraw at any time without any impact on the quality of their usual medical care.
- Is Informed About Potential Risks and Benefits: We will explain any potential risks, benefits, or discomforts associated with the study in plain language. Participants will be fully informed about what to expect, including any possible side effects related to treatments being studied.
- Is Assured of Confidentiality: Participants will be informed of how their information will be kept secure and used strictly for research purposes. Personally identifiable information will remain confidential and protected throughout the study.
- Has the Chance to Ask Questions: We encourage participants to ask any questions they may have about the study, the procedures, or what their involvement means. Our goal is to ensure they feel comfortable and informed before signing.

#### 2.7.4. Dietary compliance monitoring.

All participants will receive clear, written dietary instructions and regular reminders during study visits to promote adherence. Compliance will be monitored through dietary adherence questionnaires completed at each study visit throughout the study. Although perfect adherence cannot be guaranteed, any non-compliance will be documented and addressed with participants as needed. Significant deviations from the dietary instructions will be considered during the analysis phase as potential confounding factors to ensure a robust interpretation of the results.

## 3. Statistics

### 3.1 Statistical analysis

We plan to perform statistical analysis conforming to the standard methodology. Categorized data will be presented as absolute count and relative frequencies. Continuous data will be reported as mean and standard deviation in case of normal distribution and as median and inter-quartile range (IQR) in case of a non-normal distribution. Two-sided tests are considered to be significant with a p-value <0.05.

Analyses of continuous variables will be performed using parametric and non-parametric tests (paired t-test/ Mann-Whitney-U-test). The primary endpoint is confirmatory and is the reduction of oxalate in the Verum vs. Placebophase, i.e., Dapaglifozine vs. placebo which will be compared with a two sided-t-test. Intergroup comparisons (Dapaglifozine vs placebo) will be performed by an ANOVA for a crossover design on the group of patients who went through all trial phases and corrected with Bonferroni post-hoc Tests for multiple testing. Frequencies of urolithiasis will be tested with the x²-test, a in comparison of both treatments by the McNemar-Test. Correlations between variables will be performed using Pearson or Spearman coefficients.

Findings will be corrected for multiple testing using the Tukey method if the statistical analysis is performed with a one-way ANOVA or with the Shaffer correction if the statistical analysis is performed with a Kruskal-Wallis test. All other statistical tests will be corrected using the Bonferroni-Holm method.

### 3.2 Recruitment process

All potential study participants of the exploratory phase will be recruited according to the inclusion/exclusion criteria depending on their group: the participants with diabetes at the Div. of Endocrinology, and the cardiomyopathic participants at the Div. of Cardiology of the MUW/General Hospital of Vienna. All potential study participants of the main trial will be recruited according to the inclusion/exclusion criteria at the Department of Urology MUW/General Hospital of Vienna. The planned study procedures in the current prospective observational project are all embedded in the standard of care procedures. The earlier-mentioned study visits will be used to collect blood and urine samples. No further visits are needed except if clinically indicated. According to the standard of care, all patients with renal colic, episodes of urolithiasis, unexplained loss of kidney function, and/or haematuria will be instructed for unplanned, additional ambulance visits. In the course of this ambulance visit additional blood/urine samples will not be included in the data processing.

### 3.3 Work plan

**Fig 2:**
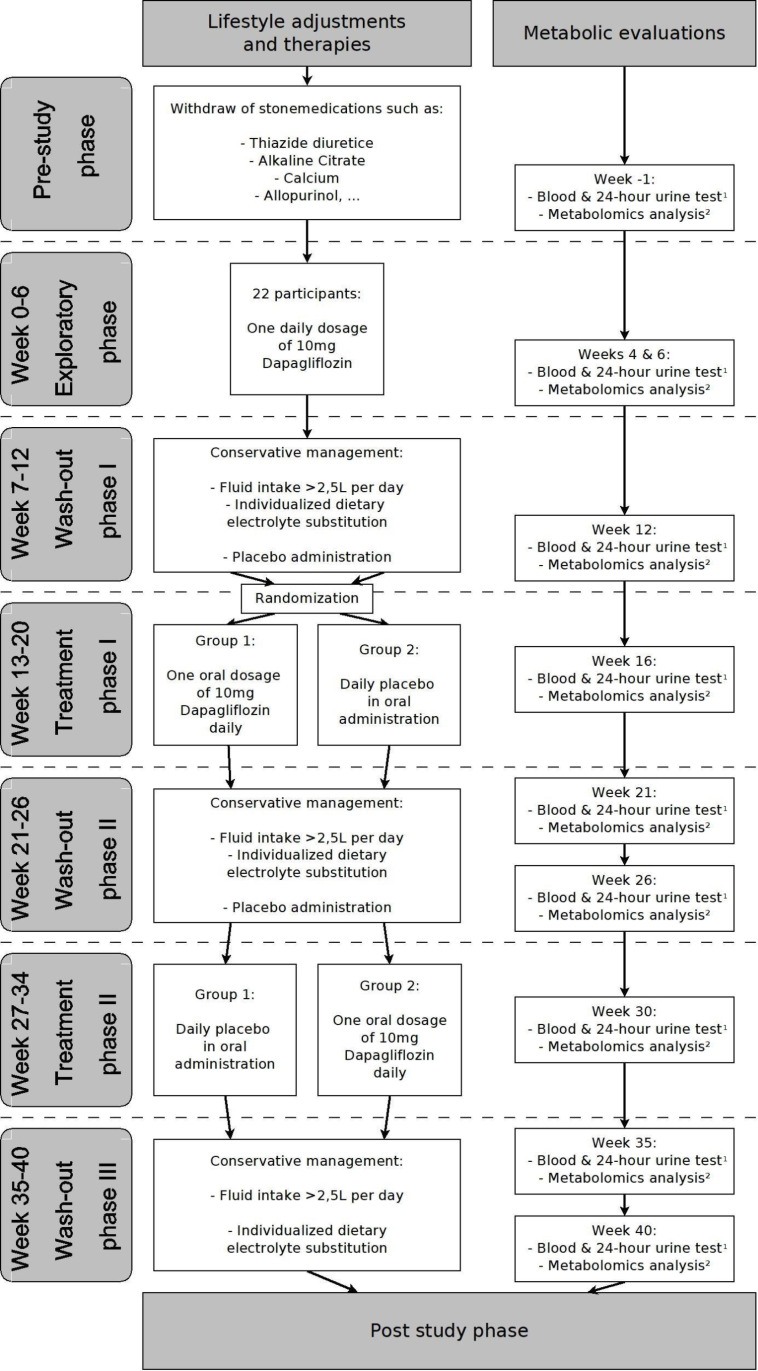
Proposed work plan; 1) Blood and 24-hour urine tests including oxalate and citrate analysis, 2) Metabolomics analysis out of blood and urine samples.

The workflow is organized into eight work packages (Fig 2).

#### WP1. Screening (Pre-study phase, 4 Months).

**Objective:** WP1 includes a prospective analysis of all patients of the Division of Cardiology of the General Hospital of Vienna with a diagnosed cardiomyopathy, diabetes mellitus II and no established SGLT-2 inhibitor therapy. Furthermore, also patients of the Department of Urology of the General Hospital of Vienna with at least one event of urolithiasis and a high risk of reoccurrence will be screened in preparation for the main trial. Baseline data as mentioned earlier of all study participants of the exploratory phase are collected **Methods:** 22 potential study participants fulfilling defined exclusion and inclusion criteria and willing to participate in the exploratory phase will be recruited. Pre-screening of potential participants of the main trial.

#### WP2. Exploratory phase (week 1–6).

**Objective** Data targeting the pathophysiology of urolithiasis and metabolomic data as mentioned earlier of a group of 22 participants will be collected. **Methods:** the group will receive an oral dose of 10mg of dapagliflozin daily; and blood- and 24h-urine screenings in weeks 4 and 6.

#### WP3: Recalculation and randomization.

**Objective** After analysing the effect size of dapagliflozin on the oxalate excretion from the date of the exploratory phase the needed number of participants for the main trial will be calculated. The WP1 already screened patients that are willing to participate will be randomized. **Methods:** effect and group size calculation as well as randomization of the patients of the main trial

#### WP4. Wash-out phase I (week 7–12).

**Objective** Baseline data as mentioned earlier of all study participants are collected. **Methods:** Blood- and 24h-urine screenings in week 6 (respectively week 12 overall) to obtain baseline data of all subjects; daily oral placebo administration in preparation for treatment phase I.

#### WP5. Treatment phase I (week 13–20).

**Objective** dapagliflozin therapy or placebo administration. **Methods:** all patients will receive an oral dose of 10mg of dapagliflozin or oral placebo daily according to their randomization; blood- and 24h-urine screenings in week 4 (respectively week 16 overall).

#### WP6. Wash-out phase II (weeks 21–26).

**Objective** Wash out of the pharmacologic agent used in treatment phase I and estimate treatment response. **Methods:** The same blood and 24h-urine screenings as before will be performed in wash-out phase II in weeks 1 and 6 (respectively weeks 21 and 26 overall). Daily placebo administration in preparation for treatment phase II.

#### WP7. Treatment phase II (week 27–34).

**Objective** dapagliflozin therapy or placebo administration. **Methods:** all patients will receive an oral dose of 10mg of dapagliflozin or oral placebo daily according to their randomization; blood- and 24h-urine screenings in week 4 (respectively week 30 overall).

#### WP8. Wash-out phase III (weeks 35–40).

**Objective** Wash-out of the pharmacologic agent used in treatment phase II and estimating treatment response. **Methods:** The same blood and 24h-urine screenings as before will be performed in wash-out phase III in weeks 1 and 6 (respectively weeks 35 and 40 overall).

#### WP9. Data evaluation and publication (post-study phase, months 16–24).

**Objective:** WP9 will comprise a thorough analysis of study results including the primary endpoint the reduction of the urine oxalate excretion by 25% under dapagliflozin therapy after 8 weeks. **Methods:** All data will be organized in a computer-based database. Statistical evaluations will be performed according to predefined models.

#### 3.3.1. Estimated completion dates.

- The study is scheduled to commence on 01.02.2025.
- Participant recruitment start (exploratory phase): 01.02.2025.
- Participant recruitment completion (exploratory phase): Recruitment is expected to be completed by 01.04.2025.
- Participant recruitment start (main phase): 01.07.2025.
- Data collection completion (main phase): Data collection is expected to finish by 01.07.2026., after the Final Phase follow-up period.
- Expected results: Initial results and analysis are projected to be completed by mid-2027.

## 4. Discussion

Dapagliflozin and the pleiotropic effects of SGLT-2 inhibitors may offer a new viable treatment option in urolithiasis. There are multiple lines of evidence for the biological plausibility of this approach. First registry data from Europe and Asia report reduced incidence of recurrent nephrolithiasis. Moreover, in a model with urolithiasis-induced rates a difference in oxalate excretion of around one standard deviation was shown between rats that received an SGLT-2 inhibitor and those that did not. So far there is still a lack of prospective, metabolic trials in humans, which our study will focus on. In the second step of a crossover design, we can show whether SGLT-2 inhibitors reduce oxalate excretion in humans.

If so, an immense financial burden on the health care system could be relieved. Calculations based on the increasing prevalence of risk factors, such as obesity and diabetes, predict increasing healthcare costs associated with stones in the urinary tract to rise by several hundreds of millions every year until 2030. If dapagliflozin decreases the incidence of urolithiasis a serious alternative to repetitive surgical treatment can be offered to affected patients. This will have the effect of easing the caseload and financial burden on both hospitals and insurance companies.

To gain data on the way to fully discover and understand the pathophysiology of stone forming we will also include a preliminary- as well as in-depth metabolomics analysis of blood and urine to narrow down potential biochemical pathways of both the pathophysiology and the pharmacologic pathways. As a study design we chose a randomized cross-over single-centre study to bring light to two conditions: firstly, the circumstances that lead to the significantly increased number of urolithiasis events with no known secondary causes. Secondly, if and how SGLT-2 inhibitors can improve the metabolic situation of patients at high risk for recurrent urolithiasis.

## Supporting information

S1 Table Spirit checklist. The table presents the SPIRIT (Standard Protocol Items: Recommendations for Interventional Trials) checklist, outlining the key elements required for a comprehensive clinical trial protocol. It includes specific recommendations for study objectives, methodology, interventions, participant selection, outcome measures, and statistical analysis.
(PDF)

S2 Appendix Laboratory parameters out of blood and urine samples. (PDF)
