# Supplementary material for: Study protocol for a randomized single-center cross-over study: Dapagliflozin treatment in recurring kidney stone patients
Source: PLoS One. 2025 Apr 24;20(4):e0322034. doi: 10.1371/journal.pone.0322034 (PMC12021238; doi:10.1371/journal.pone.0322034)
Supplement: S1 Table — The table presents the SPIRIT (Standard Protocol Items: Recommendations for Interventional Trials) checklist, outlining the key elements required for a comprehensive clinical trial protocol. It includes specific recommendations for study objectives, methodology, interventions, participant selection, outcome measures, and statistical analysis. (PDF) [file pone.0322034.s001.pdf]

Section/Item	Item No	Description	Provided Data
**Administrative Information**			
**Title**	1	Descriptive title identifying the study design, population, interventions, and trial acronym	Prospective pharmacological randomized cross-over single-center study on dapagliflozin in high-risk kidney stone patients
**Trial registration**	2a	Trial identifier and registry name. If not yet registered, name of intended registry	EudraCT Number: 2022-000994-13, ClinicalTrials: NCT05443932
	2b	All items from the World Health Organization Trial Registration Data Set	See EudraCT Number: 2022-000994-13
**Protocol version**	3	Date and version identifier	Version 1.9<br>20.10.2024
**Funding**	4	Sources and types of financial, material, and other support	To be determined
**Roles and responsibilities**	5a	Names, affiliations, and roles of protocol contributors	Dr.med.univ. Haris Omic (1)<br>ao.Univ.Prof. Dr. Harald Herkner, MSc (2)<br>PI: Assoc.Prof. Priv.Doz. Dr. Željko Kikić (3)<br>Assoc. Prof. Priv. Doz. Dr. Christian Seitz (3)<br>ao.Univ.Prof. Dr. Harald Herkner, MSc<br>Dr.rer.nat. Thomas Köcher (4)<br>Dr.med.univ. Michael Eder (1)<br>Dr.med.univ. Tarek Schrag (1)<br>1. Department of Internal Medicine III, Division of Nephrology and Dialysis, Medical University of Vienna, Vienna, Austria<br>2. Department of Emergency Medicine, Medical University of Vienna, Vienna, Austria

			3. Department of Urology Medical University of Vienna, Vienna Austria<br>4. Vienna Biocenter Core Facilities, Vienna Austria
	5b	Name and contact information for the trial sponsor	N/A
	5c	Role of study sponsor and funders in study design, data management, publication	N/A
	5d	Composition and roles of the coordinating centre, steering committee, data management team	N/A
**Introduction**			
**Background and rationale**	6a	Description of research question, relevant studies (published/unpublished)	Urolithiasis is a common disease with a high relapse rate. Dapagliflozin may offer a new treatment option.
	6b	Explanation for choice of comparators	Comparison between dapagliflozin and placebo to evaluate the effect on urolithiasis.
**Objectives**	7	Specific objectives or hypotheses	To determine the effect of dapagliflozin on oxalate excretion and other markers related to urolithiasis.
**Trial design**	8	Description of trial design, type of trial, allocation ratio, framework	Randomized cross-over design with 1:1 allocation.
**Methods: Participants, Interventions, and Outcomes**			

**Study setting**	9	Study settings and list of countries where data will be collected	Single-center study conducted at the General Hospital of Vienna, Austria.
**Eligibility criteria**	10	Inclusion and exclusion criteria for participants	Inclusion: High-risk calcium-oxalate stone formers; Exclusion: Age < 18, eGFR < 30 ml/min/1.73m².
**Interventions**	11a	Interventions for each group	Dapagliflozin (10mg daily) vs. placebo, crossover design.
	11b	Criteria for discontinuing or modifying interventions	Participants will discontinue if severe adverse effects occur or if requested by their physician
	11c	Strategies to improve adherence to intervention protocols	Regular adherence check through clinic visits
	11d	Relevant concomitant care and interventions permitted/prohibited	No additional nephrolithiasis-related treatments allowed during the trial period
**Outcomes**	12	Primary, secondary, and other outcomes, method of measurement	Primary: Oxalate excretion. Secondary: Calcium, magnesium, citrate, and uric acid excretion, eGFR, tolerability, QoL.
**Participant timeline**	13	Time schedule of enrolment, interventions, assessments, and visits	See detailed breakdown of washout and treatment phases (Figure 1)
**Sample size**	14	Estimated number of participants needed and how determined	Sample size: 22 participants based on power calculations for exploratory phase, including expected dropouts. Final Sample-Size calculation will be performed after the exploratory phase
**Recruitment**	15	Strategies for achieving adequate enrolment	Physician referral, patient databases, and advertising in urology departments
**Methods: Assignment of Interventions (for Controlled Trials)**			

**Sequence generation**	16a	Method of generating the allocation sequence	Computer-generated randomization sequence
**Allocation concealment mechanism**	16b	Mechanism of implementing the allocation sequence	Sequentially numbered, opaque, sealed envelopes to conceal allocation sequence
**Implementation**	16c	Who will generate the allocation sequence, enrol, and assign	The allocation sequence will be generated by a statistician. Study personnel will enroll participants and assign interventions
**Blinding (masking)**	17a	Who will be blinded after assignment	Participants and outcome assessors (study personnel) will be blinded
	17b	Circumstances for unblinding	Serious adverse events or upon participant request
**Methods: Data Collection, Management, and Analysis**			
**Data collection methods**	18a	Plans for assessment, collection, outcome, baseline data	Blood and urine samples collected at multiple time points, eCRF
	18b	Plans to promote retention and complete follow-up	Retention strategies, including regular follow-up calls
**Data management**	19	Plans for data entry, security, and storage	Data will be entered into a secure database, with double data entry and range checks for quality control. Data will be stored within password protected PC of the University Clinic of Urology
**Statistical methods**	20a	Statistical methods for primary and secondary outcomes	Paired t-test, ANOVA, and Bonferroni corrections.
	20b	Methods for any additional analyses	N/A
	20c	Definition of analysis population and handling of missing data	Last Observation Carried Forward (LOCF)
**Methods: Monitoring**			

**Data monitoring**	21a	Composition of data monitoring committee (DMC)	IRB of the Medical University of Vienna
	21b	Interim analyses and stopping guidelines	No interim analysis was planned.
**Harms**	22	Plans for reporting adverse events	Adverse events will be reported to the IRB within 24 hours.
**Auditing**	23	Procedures for auditing trial conduct	An independent audit will be conducted by IRB of the Medical University of Vienna at least once per year."
