# Supplementary material for: Study protocol for a randomized single-center cross-over study: Dapagliflozin treatment in recurring kidney stone patients
Source: PLoS One. 2025 Apr 24;20(4):e0322034. doi: 10.1371/journal.pone.0322034 (PMC12021238; doi:10.1371/journal.pone.0322034)
Supplement: S2 Appendix — (PDF) [file pone.0322034.s002.pdf]

Laboratory parameters out of blood and urine samples:

Blood-samples: sodium (mmol/l) potassium (mmol/l), calcium (mmol/l), magnesium (mmol/l), chloride (mmol/l), phosphorus (mmol/l), bilirubin total (mg/dl), direct bilirubin (mg/dl), indirect bilirubin (mg/dl), creatinine (mg/dl), glucose (mg/dl), Hba1c (%), lactate (mmol/l), ammoniac (µmol/l), urea (mmol/l), uric acid (µmol/l), total protein (mg/dl), albumin (mg/dl), C-reactive protein (mg/l)

Urine-samples: creatinine (mg/dl), protein (mg/dl), protein/creatinine-ratio (mg/g), specific gravity (mg/ml), pH (mmol/l), leukocytes (G/ml), haemoglobin (g/l), nitrite (mg/l), glucose (mmol/l), ketone bodies (mg/dl), calcium (mmol/l), magnesium (mmol/l), citrate (mg/l), oxalate (mg/day) amino acids (α-Aminobutyric acid, asparagine, ethanolamine, isoleucine, methionine, phenylalanine, serine, tryptophan, valine)

Metabolomic parameters derived from blood samples: Glyoxylate and dicarboxylate metabolism, Glycine, serine and threonine metabolism, Glutathione metabolism, Phenylalanine metabolism, Phenylalanine, tyrosine and tryptophan biosynthesis, Beta-alanine metabolism, Citrate cycle (TCA cycle), Glycerophospholipid metabolism, Histidine metabolism, Aminoacyl-tRNA biosynthesis, Nitrogen metabolism, Pyrimidine metabolism, Purine metabolism, Cysteine and methionine metabolism, Cyanoamino acid metabolism, d-glutamine and d-glutamate metabolism, Thiamine metabolism, Nicotinate and nicotinamide metabolism, Porphyrin and chlorophyll metabolism, Lysine degradation, Arginine and proline metabolism, Sphingolipid metabolism, Tyrosine metabolism, Tryptophan metabolism, Sulfur metabolism, Pentose phosphate pathway, Starch and sucrose metabolism, Valine, leucine and isoleucine biosynthesis, Selenoamino acid metabolism, Galactose metabolism, Glycolysis or gluconeogenesis, Propanoate metabolism, Inositol phosphate metabolism, Fructose and mannose metabolism, Butanoate metabolism, Vitamin B6 metabolism, Terpenoid backbone biosynthesis, Biotin metabolism, Glycerolipid metabolism, Synthesis and degradation of ketone bodies

Metabolomic parameters derived from urine samples

Amino acids (α-Aminobutyric acid, asparagine, ethanolamine, isoleucine, methionine, phenylalanine, serine, tryptophan, valine), creatinine, citrate, glycine, hippurate, histidine, creatine, 3-indoxylsulfate, ethanolamine, serine
